# Multicomponent Gas Standards for Hydrogen Purity Analysis According to ISO 14687 Grade D

**DOI:** 10.1021/acs.analchem.5c06763

**Published:** 2026-03-03

**Authors:** Verena Reiter, Lea A. Brandner, Sebastian Scheikl, Maurizio Tintori, Thomas Stöhr, Stefan Brandstätter, Alexander Trattner

**Affiliations:** † 27253 HyCentA Research GmbH, Inffeldgasse 15, Graz A-8010, Austria; ‡ Societa’ Italiana Acetiliene e Derivati S.I.A.D. S.p.A., via S. Bernardino, 92, Bergamo I-24126, Italy; § Graz University of Technology, Institute of Thermodynamics and Sustainable Propulsion Systems, Inffeldgasse 19, Graz A-8010, Austria

## Abstract

ISO 14687 sets strict impurity limits for hydrogen used across a wide range of applications, including fuel cell road vehicles (Grade D). Ensuring compliance with these thresholds requires precise analytical methods and certified gas standards for each regulated contaminant. However, preparing and maintaining individual reference standards for routine analysis is resource-intensive. To support the use of multicomponent standards in hydrogen purity control, this study examines the time-dependent stability of commercially prepared standards of ISO 14687 Grade D contaminants in hydrogen matrices. Using ion–molecule reaction, electron-impact mass spectrometry, along with Fourier-transform infrared spectroscopy, results show that several inert and semireactive species, including helium, argon, methane, propane, carbon dioxide, carbon monoxide, carbonyl sulfide, and halogenated compounds, remain stable for over 6 months. Ammonia concentrations stabilized after a 60-day equilibration period of the gas mixture, while oxygen showed inconsistent behavior. Formaldehyde and formic acid exhibited complete signal loss, indicating poor stability in multicomponent mixtures. These findings support the development of reliable multicomponent gas standards and strengthen hydrogen quality control practices under ISO 14687.

## Graphical abstract

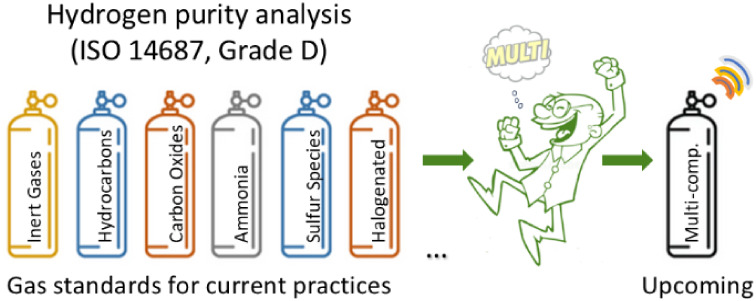

## Introduction

Hydrogen quality is a critical factor determining its suitability for various applications and, consequently, its market value. While commercial purity grades such as “5.0” or “3.8” specify only maximum total impurity levels, ISO 14687 outlines the admissible limits of individual contaminants and analytical requirements for different use cases.^1^ This is particularly relevant in the context of emerging hydrogen applications, where stringent purity requirements are essential to ensuring system reliability and performance. For example, ISO 14687 is frequently cited in technical guidelines and regulations for hydrogen infrastructure and transport via pipelines.^2–5^ In addition, it sets strict impurity limits for hydrogen used in fuel cell electric vehicles (FCEVs), which play a central role in the decarbonization of transportation and stationary power sectors.^6–9^ Reports show that even trace levels of certain impurities can significantly reduce the lifetime and efficiency of proton exchange membrane fuel cell (PEMFC) stacks through irreversible catalyst poisoning or blockage of electrode active sites, among others.^10–13^

To protect fuel cell systems from these degradation mechanisms^14,15^ and ensure H_2_ compatibility with PEMFC technology,^16^ analytical verification and quality control at trace contaminant levels are required. However, several challenges arise for reference laboratories:

(i) The threshold levels for individual impurities of ISO 14687 grade D range from 4 ppb to 300 ppm, necessitating highly sensitive and robust measurement techniques capable of reliably detecting a wide range of analyte concentrations;^17^
(ii) The impurities exhibit diverse physical properties, necessitating the use of various detection methods to accurately quantify each regulated contaminant;^18,19^

(iii) The entire process, from sampling to analysis, must be exceptionally pure, reliable, and reproducible.^20,21^

In addition, accurate quantification requires the calibration of each analyte using certified reference gases. This practice, while necessary, can be cumbersome for routine analysis due to its high costs, limited shelf life, extensive storage requirements, and the logistical effort involved in frequent cylinder changes. Multicomponent gas standards offer a promising alternative, potentially reducing this overhead. However, their broader adoption is hindered by limited knowledge of the long-term stability of individual contaminants in complex matrices and possible cross-interactions between analytes. While previous studies have focused on the sampling process, ensuring that refueling station operators can send representative H_2_ samples for purity analysis,^22^ the commercial preparation and laboratory use of multicomponent gas mixtures for quality assurance have not yet been addressed.

In this study, we investigate the stability of multicomponent gas standards for ISO 14687 grade D purity analysis under realistic market preparation and laboratory conditions. Gas mixtures of ISO 14687 grade D contaminants were commercially prepared, and individual contaminant concentrations were monitored over 6 months using Fourier-transform infrared spectroscopy (FTIR) and ion–molecule reaction/electron impact mass spectrometry (IMR-EI-MS) in accordance with ISO 21087 requirements.^23^ The findings support the wider adoption of multicomponent standards and contribute to more efficient hydrogen quality control practices.

## Experimental Section

### Preparation of Gas Mixtures

Multicomponent gas mixtures were prepared by SIAD, a specialty gas manufacturer from Italy (Bergamo), as detailed in Table 1. Standards A and B were prepared in 10-L aluminum cylinders (Luxfer Gas Cylinders), while Standards C and D were prepared in 2.5-L aluminum cylinders (Luxfer Gas Cylinders). Constituents were chosen in accordance with ISO 14687 grade D contaminants and introduced at concentrations similar to the specified threshold levels using a gravimetric preparation approach:

(a) Direct weighing: Gases are dispensed directly into the cylinder to be filled, with mass measurements made to ensure precise quantities.
(b) Indirect weighing: Gases are initially dispensed into a specialized microcylinder of small capacity and then transferred into the primary cylinder.
(c) Liquid injection by indirect weighing: A liquid is first dosed into a specialized glass capillary and then transferred into the cylinder.
(d) Weighing a gas premix directly into the standard cylinder: A premix containing the desired gases is weighed and then directly transferred into the standard cylinder.
(e) Weighing a gas premix into a smaller cylinder: A premix containing the desired gases is weighed into a smaller-capacity cylinder, which is then used to transfer the mixture into the final cylinder.

**Table 1:** Overview of the Gas Mixtures Utilized Along with Their Standard Codes and Composition

Standard			Representative ISO 14687 components														
Code	Carrier gas	H_2_O	O_2_	N_2_	He	Ar	CH_4_	C_3_H_8_	CO_2_	CO	NH_3_	COS	HCHO	HCOOH	Cl_3_C_2_H	Cl_2_C_2_H_4_	C_2_Cl_3_F_3_
A	H_2_		x	x	x	x	x	x	x	x	x	x	x	x	x	x	x
B	H_2_		x	x	x	x	x	x	x	x	x	x			x	x	x
C	H_2_												x				
D	H_2_													x			

Standard A was prepared using N_2_, He, Ar, O_2_, CH_4_, CO_2_, CO, NH_3_, formaldehyde (HCOH), and formic acid (HCOOH) at their specified limit concentrations unless otherwise noted, with hydrogen (H_2_) as the carrier gas. Then, to reduce complexity, propane (C_3_H_8_) was introduced as a model substance for total hydrocarbons, carbonyl sulfide (COS) was used to represent total sulfur compounds, and trichloroethene (Cl_3_C_2_H), 1,2-dichloroethane (Cl_2_C_2_H_4_), and Freon 113 (ClF_3_C_2_) were selected for the group of halogenated compounds.

Other compounds, in particular H_2_S, are also highly relevant for verifying compliance with ISO 14687 grade D requirements and for assessing potential impacts on fuel cell durability. However, the reliable quantification of trace-level H_2_S at sub-ISO levels using the applied FTIR methodology (vide infra) is analytically challenging due to its weak infrared absorption signature,^24^ often requiring preconcentration or chemical conversion to achieve sufficient sensitivity.^25^ To consider these methodological constraints, this study focused on COS as a representative sulfur compound, while H_2_S stability in multicomponent gas standards will be assessed in future investigations using approaches suited for trace-level H_2_S.^22^

For standard B, the same set of components was used as in standard A, except that formaldehyde and formic acid were excluded. Standards C–D were prepared to investigate these two compounds specifically and contained formaldehyde (C) and formic acid (D) in H_2_ carrier gas.

We note that standards were prepared following ISO 14687:2019, in which formic acid is individually specified, whereas in the updated standard, it is included within the constituent class of total hydrocarbons, excluding methane.

### Analytic Methods

After gas mixture preparation, the cylinders were rolled for several hours to ensure homogenization and prevent concentration gradients. Then, the contaminants were first quantified by SIAD, and the analytic amounts were shared as reference values (see Table S1 in the Supporting Information). For the subsequent stability study, the standards were shipped to HyCentA Research GmbH, where they were analyzed at the Boltzmann gas analysis laboratory over extended time periods at a constant storage temperature of 25 °C. Specifically, standard A was analyzed on days 56, 57, 58, 59, 63, 70, 79, 86, 91, 98, and 160 (MEA1-11) after gas mixture preparation. Standard B was analyzed on days 144, 145, 146, 147, 153, 160, 167, 174, 181, 188, and 208 after gas mixing, and standards C and D, containing only HCOH and HCOOH, were analyzed on days 84 and 83 after initial standard production, respectively. Precise dates for gas mixing and analysis are provided in Table S2 in the Supporting Information.

Analyte quantification at the Boltzmann laboratory was performed using the following analytic techniques: H_2_O, CO, CO_2_, COS, CH_4_, NH_3_, formaldehyde (HCHO), formic acid (HCOOH), and Freon 113 were analyzed using a Fourier Transform Infrared (FTIR) spectrometer (Bruker, USA). The FTIR system is equipped with a mercury–cadmium–telluride (MCT) detector, which is cooled with liquid nitrogen and features a Rocksolid interferometer with a permanent alignment. It uses a 5-m multireflection gas cell, with an aluminum body coated in nickel, zinc selenide (ZnSe) windows, and gold-coated mirrors. The gas cell is maintained at a temperature of 25 °C. The spectral resolution is below 0.5 cm^–1^, with a spectral rate of 0.5 cm^–1^/s (one spectrum per second). The FTIR’s housing, containing the beam path outside the gas cell, is enclosed in a Zarges box and is continuously purged with nitrogen at a flow rate of 800 mL/min. This purging creates an environment in the beam path that is free of water vapor and carbon dioxide. The FTIR system is designed to provide high-precision quantification of gaseous compounds, even at very low concentrations, without requiring calibration. The detection of analytes is achieved using reference spectra provided by the manufacturer (Bruker), which are generated under specific conditions (temperature, pressure, and flow rate). The intensity of the spectra changes linearly with the concentration, according to the Lambert–Beer law.^26^ The lower detection limits (LDLs) for the FTIR measurements were defined based on empirical values derived from prior experience. These values were subsequently confirmed through the results of an interlaboratory comparison.^17^

O_2_, He, N_2_, Ar, propane, CH_4_, COS, CO_2_, NH_3_, trichloroethene, and 1,2-dichloroethane were analyzed using a CombiSense (V&F Analyse- und Messtechnik GmbH, Austria). The CombiSense is a combined system that integrates an Electron Impact Mass Spectrometer (EI-MS) and an Ion–Molecule Reaction Mass Spectrometer (IMR-MS) within a single analyzer (IMR-EI-MS). It employs different ion sources for specific compounds such as Mercury (Hg), Xenon (Xe), and Krypton (Kr). A detailed description of the instruments and methods is provided in Table 2.

**Table 2:** Overview of the Equipment Used for ISO 14687 Contaminant Analysis

Constituentsaccording to ISO 14687:2019	Analytic technique	Lower detection limit (LDL) [ppm]	Spectral range FTIR [cm^–1^]	Ion source mass spectrometer
**Water (H**_**2**_ **O)**	FTIR	1	1611 – 1737	
**Nitrogen (N**_**2**_)	EI MS	1.07	-	e-
**Helium (He)**	EI MS	2.14	-	e-
**Argon (Ar)**	EI MS	0.12	-	e-
**Oxygen (O**_**2**_)	IMR MS	0.25	-	Xe
**Methane (CH**_**4**_)	FTIR/IMR MS	0.018	1231.8–1329.8	Xe
**Propane**	IMR MS	0.042	-	Xe
**Carbon dioxide (CO**_**2**_)	FTIR/IMR MS	<1	2279.4–2394.7	Kr
**Carbon monoxide (CO)**	FTIR	0.05	2142.4–2211.4	
**Ammonia (NH**_**3**_)	FTIR/IMR MS	0.03	1042.5–1126.3	Hg
**Carbonyl sulfide (COS)**	FTIR/IMR MS	0.01	2020–2089	Xe
**Formaldehyde (HCHO)**	IMR MS/FTIR	0.002	1070–1140	Xe
**Formic acid (HCOOH)**	FTIR	0.02	2732–2836	Xe
**Trichloroethene (Cl**_**3**_ **C**_**2**_ **H)**	IMR MS	0.004	-	Xe
**Dichloroethane (Cl**_**2**_ **C**_**2**_ **H**_**4**_)	IMR MS	0.001	-	Hg
**C**_**2**_ **Cl**_**3**_ **F**_**3**_ **(Freon 113)**	FTIR	0.01	1150–1199	-

The full measurement setup, including FTIR and IMR-EI-MS, was optimized with the shortest feasible gas paths. It consists of permanently sealed 1/4″ and 1/8″ pipes made of SS 316 L. These are coated with SilcoNert 2000 as an inert, nonreactive silicon coating. The measuring setup is permanently pressurized with high-purity hydrogen (min. 5.0 by Linde) so that no atmospheric impurities can accumulate in the lines. Before analysis, the measuring setup is flushed with high-purity hydrogen and passed through a purification column (ZPure PolyGas I 130 cm^3^), installed before the instrumental section. Purging of the system is performed until all impurities are completely removed. During purging, an FTIR raw measurement is performed to check the effectiveness of the process. When the system is sufficiently purged (detected water content <0.8 ppm), a background measurement is taken with the FTIR. Afterward, the instrument is set to measure transmission spectra. In parallel, the mass spectrometer is calibrated with the calibration gases listed in Table S3 and Table S4 in the Supporting Information. For IMR-EI-MS, a background calibration is done with 7.0 hydrogen (Linde). Afterward, span calibration is performed with all calibration gases in succession. The calibration process is fully automated and performed each day prior to the start of measurement activities.

Once both FTIR and mass spectrometry are calibrated, the standard cylinder is connected to the measurement pathway, and the connecting pipes are purged using pressure cycling through the vent. After the purging process is complete, the standard is opened to the measuring path and analyzed using both analyzers in parallel. The measurement is conducted using a flow of 500 mL/min (MS) and 1 L/min (FT-IR) for a minimum of 20 min.

Data processing and analysis were carried out using the respective software tools: OPUS GA for the FTIR measurements and V&F Client 2.5 for the IMR-EI-MS data. Mean values were calculated from data points after stabilization of the signal, and statistical error is expressed as three times the standard deviation, as previously described.^27^

To assess the comparability of measurements between SIAD and HyCentA’s Boltzmann laboratory, the results for MEA1 of standard A were additionally evaluated using the zeta-score method in accordance with ISO 13528:2022.^28^ This method describes the relative deviation from the reference value provided by SIAD and is interpreted as follows: |ζ| ≤ 2 indicates a satisfactory result, 2 < |ζ| ≤ 3 is considered questionable, and |ζ| > 3 is deemed unsatisfactory. The zeta-score is calculated according to eq 1:

1 $$\zeta_{i}=\frac{x_{i}-x_{\mathrm{RV}}}{\sqrt{u^{2}\left(x_{i}\right)+u^{2}\left(x_{\mathrm{RV}}\right)}}$$

with *x*_i_ representing the result of MEA1 of standard A, *x*_RV_ representing the reference value provided by SIAD, *u*(*x*_i_) representing the standard uncertainty of the result of MEA1 of standard A, and *u*(*x*_RV_) representing the standard uncertainty of the reference value provided by SIAD.

## Results and Discussion

### Time-Resolved Stability Analysis of Mixed 14687 Grade D Components in H_2_ Carrier Gas

A multicomponent gas standard (standard A) was prepared by SIAD with N_2_, He, Ar, O_2_, CH_4_, propane, CO_2_, CO, NH_3_, COS, formaldehyde, formic acid, trichloroethene, 1,2-dichloroethane, and Freon 113 mixed at their maximum ISO 14687:2019 grade D concentrations unless otherwise indicated. To assess if these analytes could be reliably quantified at their reported levels, we first performed zeta-score evaluation in compliance with ISO 13528:2022. For this, standard A was analyzed immediately upon arrival at the Boltzmann laboratory using combined FTIR and IMR-EI-MS detection methods—an analytic approach proven effective for ISO 14687 contaminant analysis.^17^ The obtained results were then compared to the reference values provided by SIAD, with the exception of N_2_, which was not individually reported by the manufacturer. We further note that at the time of the gas analysis at the Boltzmann laboratory, 8 weeks had passed since the gas preparation and 7 days since the initial analysis by SIAD. Thus, any degradation events during these time periods cannot be accounted for. However, these timelines reflect typical market conditions and are thus representative of real-world lead times for supplying multicomponent gas standards.

The zeta-scores obtained by the Boltzmann laboratory show that for He, Ar, O_2_, CH_4_, CO_2_, CO, COS, trichloroethene, and 1,2-dichloroethane, data points are within a zeta score of 3, indicating reliable measurement of these analytes compared to the reference values (Figure 1a). However, for propane (ζ = 8.3), ammonia (ζ = −4.0), and Freon 113 (ζ = 38.9), results were outside the depicted boundaries (green boundaries (|ζ| ≤ 2), red boundaries (|ζ| ≤ 3)). In the case of propane, the deviation was observed in the positive direction, indicating an overestimation of the analyte present in the gas mixture. The authors hypothesize that this is likely linked to analytic variability of the daily calibration process, rather than potential degradation events, which typically appear as a deviation in the negative direction.^29^ For ammonia, the underestimation could be attributed to potential degradation of the analyte as previously reported.^22^ On the other hand, for Freon 113, the observed difference exceeds expected analytical variability (Figure 1b), so that a systematic bias, either in the measured or the reference value or outside standard contamination, appears likely. Indeed, analysis of standard B showed different results for Freon 113, as discussed in section Long-Term Stability of Multicomponent Gas Mixtures (vide infra).

**Figure 1:**
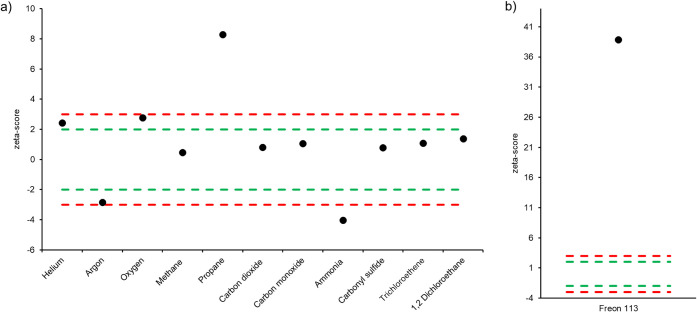
**a-b)** Zeta-scores obtained for the different analytes of standard A.

Notably, formaldehyde and formic acid could not be detected when analyzing standard A at the Boltzmann laboratory. This indicates degradation of the analytes (formally 0.100 ppm formaldehyde, 0.200 ppm formic acid, as reported by SIAD) in the H_2_ carrier gas during the 56 days since gas mixing, and aligns with limited stabilities of low amount fractions of formaldehyde in hydrogen gas observed in the literature.^29^ To investigate whether the instability of formaldehyde and formic acid arises from interactions with other ISO 14687 components or the hydrogen carrier gas itself, additional standards were prepared, containing only formaldehyde (standard C) and formic acid (standard D) in the hydrogen carrier gas, respectively. After storage at 25 °C for 84 days (C) and 83 days (D), formaldehyde, originally present at a reference concentration of 0.116 ppm, was no longer detectable by FTIR analysis, suggesting full degradation of formaldehyde in the hydrogen matrix. In the case of formic acid, a concentration decrease from 0.201 ppm (reference value) to 0.105 ± 0.027 ppm was observed, indicating slightly better analyte stability compared to the complete loss of signal observed in the multicomponent mixture (standard A). However, the observed 52% decrease in formic acid concentration contrasts with previous studies, which reported only minor losses of formic acid due to adsorption on gas cylinder surfaces and generally suggested shelf lives in hydrogen of up to 1 year.^30^ We hypothesize that the observed discrepancy is most likely attributed to differences in the gas cylinder materials. A more detailed discussion on this topic is provided in section Comparison of Cylinder Materials for Long-Term Applications.

Next, we studied the time-dependent stability of the fuel gas contaminants in standard A. Table 3 shows the analytical values and standard deviation obtained after 56 (MEA1) and 160 days (MEA11), as well as the mean quantified amount over the complete testing period (Mean). Additionally, the results of all data points are depicted in Figure 2, with 10% thresholds of the reference value indicated as gray dotted lines, serving as a monitoring criterion to assess the stability of the analyte concentrations over time. The mean amount quantified by the Boltzmann laboratory over the full analysis period is depicted as an orange line. Numerical values and standard deviation for all measurement points are provided in Table S5 in the Supporting Information.

**Table 3:** Overview of Reference Values and Measurement Data of Standard A

			RV		MEA 1		Mean over 160 d	
Constituents	Method	Unit	Value	*u*	Value	*u*	Value	*u*
**Water**	**FTIR**	**ppm**			29.881	2.928	31.635	1.298
**Nitrogen**	**EI MS**	**ppm**	-	-	5511.800	10.100	5541.361	27.975
**Helium**	**EI MS**	**ppm**	340.000	7.000	357.240	1.310	347.925	1.261
**Argon**	**EI MS**	**ppm**	303.000	7.000	282.620	1.610	283.378	0.586
**Oxygen**	**IMR MS**	**ppm**	6.700	1.000	9.603	0.340	9.521	1.330
**Methane**	**FTIR**	**ppm**	101.500	4.500	101.116	12.748	102.880	1.327
**Propane**	**IMR MS**	**ppm**	1.950	0.100	3.034	0.085	2.481	0.068
**Carbon dioxide**	**FTIR**	**ppm**	2.090	0.110	2.130	0.256	2.166	0.028
**Carbon monoxide**	**FTIR**	**ppm**	0.190	0.020	0.208	0.026	0.207	0.004
**Ammonia**	**FTIR**	**ppm**	1.050	0.020	0.847	0.178	0.867	0.097
**Carbonyl sulfide**	**FTIR**	**ppm**	0.054	0.005	0.057	0.007	0.058	0.001
**Formaldehyde**	**FITR**	**ppm**	0.100	0.020	-	-	-	-
**Formic acid**	**FITR**	**ppm**	0.200	0.030	-	-	-	-
**Trichloroethene**	**IMR MS**	**ppm**	0.045	0.005	0.053	0.006	0.053	0.014
1,2-Dichloroethane	**IMR MS**	**ppm**	0.051	0.005	0.061	0.006	0.059	0.011
**Freon 113**	**FTIR**	**ppm**	0.045	0.005	0.309	0.026	0.297	0.014

**Figure 2:**
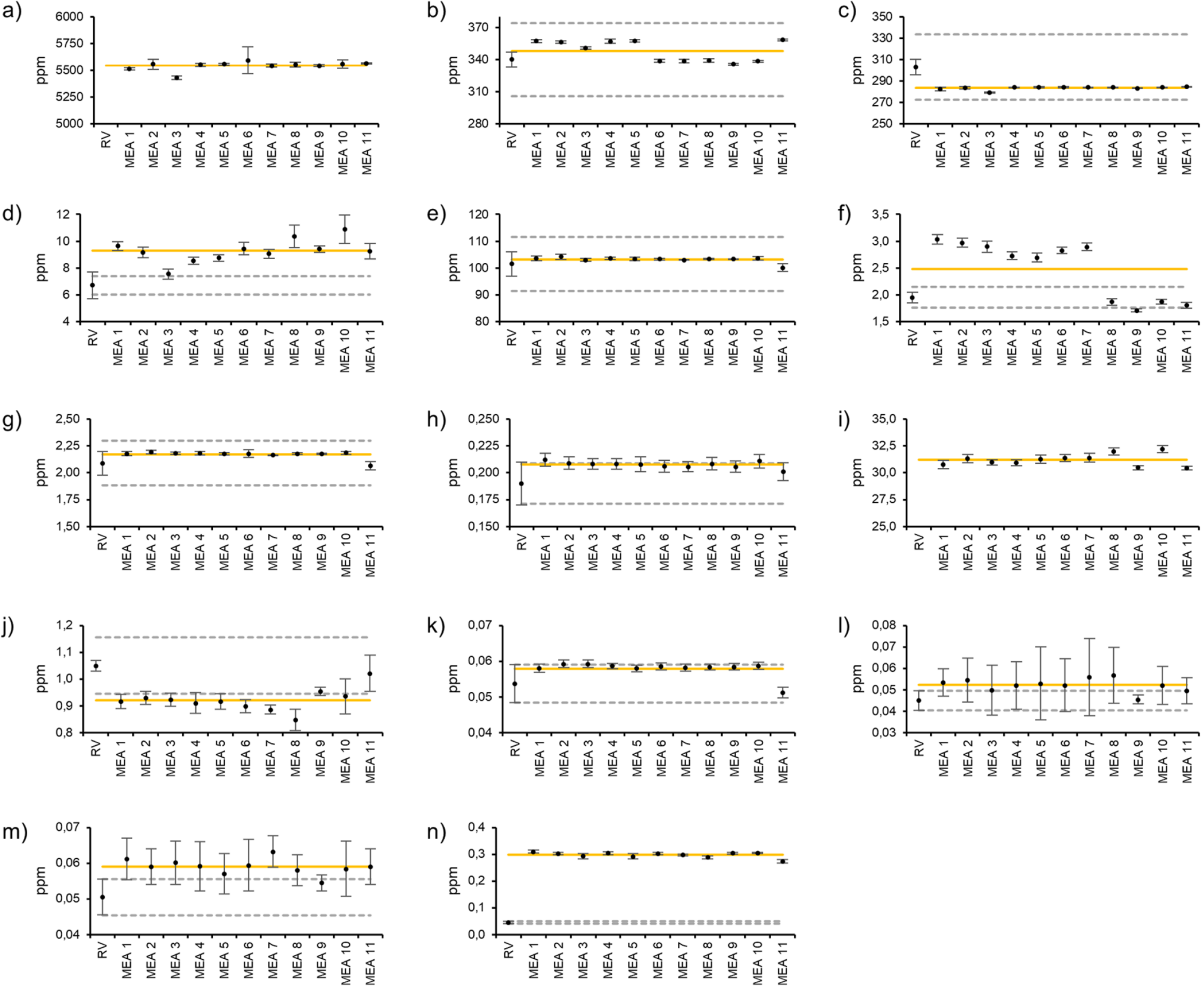
ISO 14687 analyte concentrations of standard A over a 160-day time period at different measurement points (MEA1-11) for **a)** N_2_, **b)** He, **c)** Ar, **d)** O_2_, **e)** CH_4_, **f)** propane, **g)** CO_2_, **h)** CO, **i)** water, **j)** NH_3_, **k)** COS, **l)** trichloroethene, **m)** 1,2-dichloroethane, and **n)** Freon 113.

The analytical results for N_2_, He, Ar, CH_4_, CO_2_, CO, and COS remain within 10% of the respective reference values and exhibit measurement uncertainties below 10%. No consistent upward or downward trends are observed over the 160-day period, indicating that these analytes are stable over extended durations and are not affected by interactions with other components in the gas mixture.

For oxygen, analysis via IMR-MS consistently produced values exceeding the 10% threshold relative to the reference value. The relative variability between the measurements was found to be 9.5%, with no consistent downward or upward trend over the full investigation period, indicating no clear degradation or cross-interaction behavior. Thus, the changes in the analyte concentration are attributed to operational anomalies and require further investigation into the stability of oxygen in multicomponent gas mixtures.

For propane, concentrations were overestimated during measurements of MEA1 through MEA7. This deviation is likely due to a positive bias during the daily calibration process at the start of the study. We also note that the calibration gas was changed after MEA7, upon which values for propane stabilized within the 10% threshold of the reference value for the remaining data points (MEA8 and onward). This was also confirmed using zeta-score evaluation (see Figure S1 in the Supporting Information).

For ammonia, measured concentrations were below 90% of the reference value throughout the study period, suggesting analyte degradation or loss after initial analysis by SIAD. Unlike previous reports that observed complete signal loss linked to potential ammonia adsorption onto internal cylinder surfaces,^22^ our data show only a 13% drop between the reference value and MEA1. This indicates that the aluminum cylinders used in this study may be better suited to retain ammonia stability in multicomponent gas mixtures. Indeed, after the initial decline, the results exhibited a relative variability of only 4.7% with no consistent downward trend, indicating potential stability of the analyte after prolonged equilibration in the aluminum gas cylinder.

Both trichloroethene and 1,2-dichloroethane showed average concentrations slightly exceeding the 10% reference threshold over the full observation period. Despite this, the data appear generally stable, with individual error bars overlapping with the 10% limit of the reference value. This may reflect a slightly positive calibration bias rather than true instability or changes due to chemical cross-interactions.

While measured values for Freon 113 were consistently higher than the reference values, intrameasurement variability was low, with deviations between data points remaining below 4%. This points to a systematic error or external Freon 113 contamination during the analysis process, consistent with the previously observed zeta-score evaluation, but indicates stable behavior of the component in multianalyte gas mixtures at these concentrations.

Interestingly, H_2_O was not added as a component to standard A during gas mixing but was detected at a concentration of approximately 32 ppm across the full investigation period at the Boltzmann laboratory. While H_2_O has previously been identified as a decay product formed by the reaction of oxygen with the hydrogen carrier gas,^22^ contamination during the gas mixture preparation process cannot be fully excluded, as reference values for water were not provided by SIAD.

Combined, the results of standard A show that among the mixed gases, N_2_, He, Ar, CH_4_, propane, CO_2_, CO, and COS and the halogenated compounds are stable over the 160-day period and thus considered suitable for use in multicomponent calibration standards. The inclusion of ammonia in a mixed standard seems partially limited, as analyte loss was observed at the start of the stability analysis. For O_2_, stability in the mixed analyte matrix was shown to be challenging, with high relative variability between data points being the critical limiting factor. In the case of Freon 113, good stability was achieved over the testing period at high concentration levels of about 0.300 ppm, but stability at maximum concentration levels according to ISO 14687 (0.050 ppm) has yet to be investigated.

### Long-Term Stability of Multicomponent Gas Mixtures

For standard B, He, Ar, O_2_, CH_4_, propane, CO_2_, CO, NH_3_, COS, trichloroethene, 1,2-dichloroethane, and Freon 113 were considered in the stability analysis after gas mixing. Formic acid and formaldehyde were removed from the gas mixture to test the stability of combined analytes that have shown at least partial stability over the previous 160 day trial (standard A). Additionally, to evaluate method accuracy and precision for the measurement of the remaining gases after an extended time period, we used both FTIR and MS detection methods for CH_4_, CO_2_, ammonia, and COS. Table 4 shows the analytical values and standard deviations of standard B after 208 days of storage at 25 °C. An individual comparison of the data points is provided in Figure 3, with reference values depicted as black points, measured values after 208 days depicted as orange squares, and 10% thresholds of the reference value indicated as gray dots or dotted lines. Analytical data over the full observation period are provided in Table S6 and Figure S2 in the Supporting Information.

**Table 4:** Overview of Reference Values and Measurement Data of Standard B after 208 Days

			RV		After 208 days (MEA 10)	
Constituents	Method	Unit	Value	*u*	Value	*u*
**Helium**	**EI MS**	**ppm**	303.000	6.200	321.412	2.045
**Argon**	**EI MS**	**ppm**	303.000	6.200	312.357	0.386
**Oxygen**	**IMR MS**	**ppm**	4.200	0.400	6.080	0.327
**Methane**	**IMR MS**	**ppm**	103.000	2.100	104.851	0.994
**Methane**	**FTIR**	**ppm**	103.000	2.100	109.687	0.145
**Propane**	**IMR MS**	**ppm**	2.000	0.100	1.995	0.072
**Carbon dioxide**	**FTIR**	**ppm**	2.150	0.110	2.237	0.004
**Carbon dioxide**	**IMR MS**	**ppm**	2.150	0.110	0.812	0.123
**Carbon monoxide**	**FTIR**	**ppm**	0.189	0.016	0.208	0.006
**Ammonia**	**IMR MS**	**ppm**	1.020	0.130	0.901	0.041
**Ammonia**	**FTIR**	**ppm**	1.020	0.130	1.134	0.072
**Carbonyl sulfide**	**IMR MS**	**ppm**	0.054	0.005	0.057	0.002
**Carbonyl sulfide**	**FTIR**	**ppm**	0.054	0.005	0.060	0.001
**Trichloroethene**	**IMR MS**	**ppm**	0.050	0.005	0.047	0.005
1,2-Dichloroethane	**IMR MS**	**ppm**	0.053	0.005	0.055	0.006
**Freon 113**	**FTIR**	**ppm**	0.058	0.005	0.059	0.011

**Figure 3:**
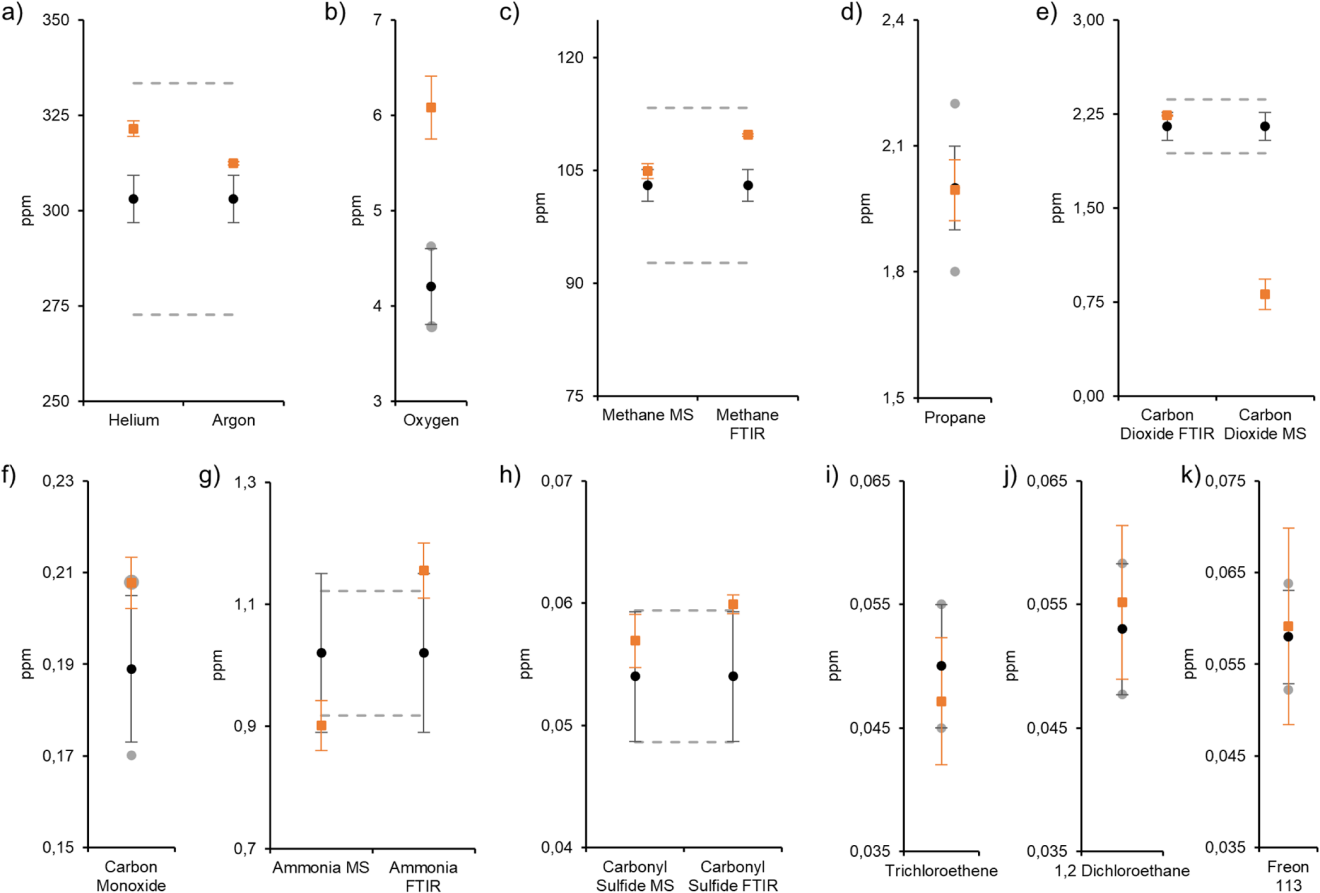
**a-k)** Comparison of reference values (black dots) and observed analyte concentrations (orange squares) of standard B after 208 days.

The stability performance of several analytes after an extended time period of 208 days confirms the trends observed in the initial stability assessment (standard A). Results for He, Ar, CH_4_, CO_2_, and CO remained within ±10% of the respective reference values, indicating stable behavior of these components over 6 months and confirming their suitability for inclusion in multicomponent calibration gas standards when supplied within typical market timelines.

For CO_2_, FTIR measurements were also in good agreement with the reference value, whereas mass spectrometric (MS) analysis tended to underestimate the concentration, suggesting matrix-related signal suppression or insufficient calibration sensitivity in the MS method.

Oxygen was again systematically overestimated in IMR-MS analysis, consistent with previous results for standard A. This persistent deviation, coupled with high variability and measurement uncertainty, underscores the challenges to reproducibly analyze O_2_ with IMR-MS in multicomponent gas mixtures.

On the other hand, propane exhibited good agreement with the reference value, similar to the measurements MEA8-11 of standard A, indicating accurate calibration and stable behavior.

For ammonia, the measured value by mass spectrometry (MS) was observed slightly below, while the measured value by FT-IR was slightly above, the ±10% threshold relative to the reference value after 208 days. Nevertheless, no significant downward trend was revealed over the 208-day period, indicating that NH_3_ remained stable within the gas mixture, with no substantial loss or degradation (see Figure S2). These findings contrast with previous results, which exhibited a maximum 20% decrease in ammonia concentration (standard A) or even complete signal loss.^22^ While further analysis is required to accurately characterize the observed variability in NH_3_ retention over time and to potentially understand underlying adsorption and desorption dynamics, we want to note that the time between gas preparation and analysis might be critical for NH_3_ stability in multicomponent mixtures. Indeed, we observed that for standard A, 49 days passed between gas preparation and analysis by SIAD, and 62 days until subsequent analysis was performed at the Boltzmann laboratory, upon which a maximum 20% drop in concentration was observed. After the 62 days, the NH_3_ concentration appeared to stabilize for the rest of the investigated time period with a variability below 5% between data points. In comparison, standard B was analyzed by SIAD 63 days after preparation, showing an NH_3_ concentration of 1.020 ppm. Then, similar values were obtained in the follow-up measurement at the Boltzmann laboratory after 208 days (1.134 ppm by FT-IR; 0.901 ppm by MS). These observations raise the question of whether an equilibrium between adsorbed and gaseous NH_3_ is reached after approximately 60 days in a gas cylinder. To confirm this hypothesis, future targeted studies, which explore and precisely quantify potential adsorption-related effects, are needed.

Carbonyl sulfide (COS) showed stable behavior, as previously observed in standard A, with the MS result remaining within the 10% reference threshold and FTIR values slightly overestimated. This minor bias in FTIR data may reflect spectral overlap or baseline effects rather than true instability, but it suggests that MS is a more reliable method for COS quantification.

All halogenated hydrocarbons, including trichloroethene, 1,2-dichloroethane, and Freon 113, were quantified within 10% of the reference values after 208 days. While the average measurement uncertainties were relatively high (between 10% and 12%), they were comparable to those of the reference values (10%), suggesting that the quantification of halogenated compounds remains analytically challenging, primarily due to the low concentration levels and the inherent constraints of the detection methods used.

Overall, the results of standard B support the findings from standard A. Several ISO 14687 grade D contaminants, including He, Ar, CH_4_, propane, CO_2_, CO, COS, and halogenated compounds, do not show a noticeable decline in stability over an increased time period of 6 months and 24 days. The results for ammonia are also promising, with measured values almost matching the reference after equilibration of NH_3_ in the multicomponent gas mixture for ca. 60 days. In contrast, for O_2_, the high relative variability between data points indicates limited suitability in mixed gas standards.

### Comparison of Cylinder Materials for Long-Term Applications

The use of multicomponent standards is intended to streamline analytical workflows and reduce laboratory overhead. Beyond this practical objective, however, the stability of analytes and their analytical recovery in gaseous hydrogen matrices are also highly relevant to other research fields, including hydrogen gas sampling and quality assurance. In this context, the material of the gas-containing vessel plays a critical role.

Both the present study and previous investigations demonstrate that the stability of many contaminants specified in ISO 14687, such as He, Ar, CH_4_, ethane, propane, CO_2_, CO, COS, and halogenated species, can be maintained over extended storage periods of up to 208 days, largely independent of the cylinder material. In contrast, significant differences in analyte stability are observed for other compounds. Notably, formaldehyde, formic acid, H_2_S, and ammonia exhibit pronounced variations in stability that seem to depend on both the vessel material and the component concentration level, as summarized in Table 5.

**Table 5:** Stability of ISO 14697-Relevant Species with Regard to Cylinder Material and Contaminant Concentration^a^ ^b^

		Time stability (days)/Stability comments				
Component	Nominal component concentration [ppm],this study and respective references	Untreated aluminum [this study]	SPECTRA-SEAL- treated aluminum^22^	SGS-finished aluminum^22^	SPECTRA-SEAL aluminum^31^	SPECTRA-SEAL-passivated aluminum^30^
Helium	340, 300/1500 ^22^	160/208	120	120	n.a^.a^	n.a.
Argon	303, 100 ^22^	160/208	120	120	n.a.	n.a.
Oxygen	6.7, 5 ^22^	< MEA1	120	120	n.a.	n.a.
Methane	101.5, 1 ^22^	160/208	120	120	n.a.	n.a.
Ethane	n.a., 0.5 ^22^	n.a.	120	120	n.a.	n.a.
Propane	1.95	160/208	n.a.	n.a.	n.a.	n.a.
Carbon dioxide	2.09, 2 ^22^	160/208	120	120	n.a.	n.a.
Carbon monoxide	0.19, 0.2,^22^ 0.11 ^31^	160/208	120	120	35	n.a.
Ammonia	1.0, 0.2 ^22^	160^b^/208	<1	<1	n.a.	n.a.
Carbonyl sulfide	0.054	160/208	n.a.	n.a.	n.a.	n.a.
Formaldehyde	0.1, 0.2 ^22^	< MEA1	<1	<1	n.a.	n.a.
Formic acid	0.2, 0.2,^22^ 4–100 ^30^	< MEA1	60	<2	n.a.	365 (730)
H_2_S	n.a., 0.007,^22^ 0.04 ^31^	n.a.	<1	120	35	n.a.
Dichloromethane	n.a., 0.05 ^22^	n.a.	120	120	n.a.	n.a.
Trichloroethene	0.045	160/208	n.a.	n.a.	n.a.	n.a.
1,2-Dichloro-ethane	0.051	160/208	n.a.	n.a.	n.a.	n.a.
Freon 113	0.045	160/208	n.a.	n.a.	n.a.	n.a.

a n.a. = not available.

b NH_3_ stable after equilibration for ca. 60 days.

Complete loss of formaldehyde was observed in the multicomponent standard (Standard A) as well as in the single-component preparation (Standard C) at concentration levels of approximately 0.1 ppm when stored in untreated aluminum cylinders. Comparable behavior has been reported in the literature: cylinders containing 0.2 ppm formaldehyde exhibited losses of 50% and 60% within 24 h in SPECTRA-SEAL-treated and SGS-finished aluminum cylinders, respectively.^22^ These findings indicate that formaldehyde is intrinsically difficult to stabilize in hydrogen matrices at concentration levels close to the limit specified in ISO 14687:2025 (0.2 ppm), largely independent of the sampling configuration or cylinder surface treatment. Additionally, previous studies have attributed formaldehyde losses to surface-mediated degradation, particularly conversion to methanol and dimethoxymethane, with cylinder walls proposed to act as catalytic surfaces for these reactions.^29^ In light of this mechanism, comparable degradation pathways are likely to occur in untreated aluminum cylinders, explaining the complete depletion observed in the present study. The absence of stabilizing surface treatments may further enhance the adsorption or catalytic activity, accelerating analyte loss at trace concentration levels.

Formic acid exhibited similarly unstable behavior in untreated aluminum cylinders. In the multicomponent mixture (Standard A), complete loss was observed after 56 days. When prepared as a single analyte in hydrogen (Standard D), only 52% of the nominal concentration remained after 83 days. Although the precise onset of degradation cannot be determined due to the time interval between preparation and the first analysis, the observed decay is consistent with literature data. In SGS-finished aluminum cylinders, complete loss of formic acid has been reported within 48 h, whereas SPECTRA-SEAL-treated systems maintained stability for approximately 2 months before gradual losses occurred.^22^ In addition to the cylinder material, both the initial concentration and mixture composition seem to affect stability. Formic acid has been reported to remain stable for more than 2 years in SPECTRA-SEAL-passivated aluminum cylinders at concentrations between 4 and 100 ppm in the absence of other contaminants.^30^ However, at lower concentrations (0.7 ppm), stability decreased within 20 days before reaching a plateau at approximately 86% of the initial concentration. This initial decline was attributed to partial adsorption of formic acid onto cylinder walls.^30^ Such adsorption effects are likely to contribute to the analyte loss in untreated and SGS-finished aluminum cylinders. Additional long-term degradation pathways, potentially involving surface-catalyzed reactions or interactions with trace impurities, remain to be clarified.

Although H_2_S was not experimentally investigated in the multicomponent standards (A and B), its relevance as a catalyst poison necessitates careful consideration in hydrogen sampling applications. Literature data demonstrate a strong dependence of H_2_S stability on the cylinder material and surface treatment. In SPECTRA-SEAL-treated aluminum cylinders, stability of less than 24 h has been reported, whereas SGS-finished aluminum cylinders exhibited significantly improved stability of up to 4 months at a concentration of 7 ppb.^22^ In another study, 40 ppb H_2_S was examined in both SPECTRA-SEAL-treated and untreated aluminum vessels in the presence of 110 ppm of CO.^31^ An initial loss of approximately 10% was observed immediately after sampling in both vessel types, followed by stable concentrations over a 5-week period. These findings further emphasize that, in addition to cylinder material, analyte concentration and the presence of cocontaminants influence stability behavior. For gas sampling at ISO 14687-relevant trace levels, the SGS finish currently appears to provide the most reliable retention of H_2_S. For multicomponent standard preparation, however, both treated and untreated aluminum cylinders may be suitable at higher concentration levels.

Ammonia exhibited comparatively stable behavior in the multicomponent mixtures stored in untreated aluminum cylinders for up to 208 days. After an initial decrease of approximately 20% within the first 62 days, measured concentrations remained within defined 10% thresholds, indicating improved long-term stability compared to literature reports for other cylinder materials. It should be noted, however, that the initial concentration in this study (approximately 1 ppm) was five times higher than the 0.2 ppm investigated in SPECTRA-SEAL-treated and SGS-finished cylinders, where complete loss was reported within 24 h.^22^ The pronounced instability at lower concentrations, compared to the comparatively stable behavior following initial equilibration at higher levels, supports the hypothesis of the partial irreversible adsorption of NH_3_ onto cylinder walls. Surface saturation effects may reduce further losses once adsorption sites are occupied.

Overall, the untreated aluminum cylinders evaluated in this study demonstrate adequate performance for the preparation and storage of multicomponent hydrogen standards, with stable retention of most species regulated under ISO 14687:2025 for up to 208 days. Instability was primarily confined to reactive compounds, particularly formaldehyde and formic acid, which exhibited pronounced losses consistent with literature reports. Ammonia showed acceptable long-term stability following an initial equilibration phase, whereas the stability of H_2_S under comparable conditions remains to be systematically investigated. Collectively, these findings indicate that untreated aluminum cylinders are suitable for multicomponent standard preparation at defined concentration levels. However, for hydrogen sampling applications at ISO 14687-relevant trace concentrations, careful selection of cylinder material and surface treatment is essential to ensure reliable contaminant stability and maintain data integrity.

## Conclusions

In this study, we investigated the long-term stability of ISO 14687 grade D contaminants in multicomponent gas mixtures in hydrogen matrices. Through comparative analysis of Standard A and Standard B, our findings demonstrate that several inert and semireactive contaminants, including He, Ar, methane, propane, carbon dioxide, carbon monoxide, carbonyl sulfide, and halogenated species, remain stable over periods of 160 to 208 days. These results support their suitability for use in multicomponent gas standards for hydrogen quality assurance. Additionally, ammonia showed promising behavior, reaching a constant concentration within approximately 60 days after gas mixing and maintaining its level thereafter.

However, challenges remain for oxygen due to the high observed relative variability in the measurements. Thus, its reliable inclusion in multicomponent calibration standards remains questionable. Additionally, the authors note that future studies exploring adsorption and desorption phenomena on different cylinder material surfaces, as well as understanding the effects of transport and storage conditions on the stability of gas mixtures, could provide further insight into this topic. Combined with our results, advancing these aspects will strengthen hydrogen quality control and support the commercial supply and reliable use of multicomponent calibration standards in practical applications.

## Supplementary Material



## Notes

Certified reference values, calibration gases, analyte concentration data (PDF)
