# Supplementary material for: Multicomponent Gas Standards for Hydrogen Purity Analysis According to ISO 14687 Grade D
Source: Anal Chem. 2026 Mar 3;98(10):7403–12. doi: 10.1021/acs.analchem.5c06763 (PMC13000869; doi:10.1021/acs.analchem.5c06763)
Supplement: Supplementary file 1 [file ac5c06763_si_001.pdf]

Supporting Information

Multi-component gas standards for hydrogen purity analysis according to ISO 14687 Grade D

Verena Reiter [a§], *Lea A. Brandner* [a§], *Sebastian Scheikl* [a], *Maurizio Tintori* [b], *Thomas Stöhr* [a], *Stefan Brandstätter* [a], *Alexander Trattner* [a,c*]

[a] HyCentA Research GmbH, Inffeldgasse 15, A-8010, Graz, Austria

[b] Societa' Italiana Acetiliene e Derivati S.I.A.D. S.p.A., I-24126 Bergamo - Via S. Bernardino, 92, Italy

[c] Graz University of Technology, Institute of Thermodynamics and Sustainable Propulsion Systems, Inffeldgasse 19, A-8010 Graz, Austria

* Email: trattner@tugraz.at

[§] V. R. and L. A. B. contributed equally to this work

Table of Contents

Table S1: Certified concentrations (reference values) of the multi-component gas mixtures (samples A-D) provided by SIAD. S3

Table S2: Detailed breakdown of the production days and analysis days of samples A-D. S4

Table S3: Calibration standards for EI-IMR-MS calibration provided by Gas Supplier: SIAD, Bergamo, unless otherwise indicated. S5

Table S4: Gas standards for enhancement calibrations used for EI-IMR-MS extended calibration. S5

Table S5: Analyte concentrations of sample A. S6

Figure S1: Zeta-score evaluation of sample A, showing a satisfactory zeta score of -0.70 for propane 86 days after gas mixing (MEA 8). S8

Table S6: Analyte concentrations of sample A. S9

Figure S2: Comparison of reference values (RV) and observed analyte concentrations of sample B at different time points (MEA1-10) in the 208-day stability investigation. S11

Table S1: Certified concentrations (reference values) of the multi-component gas mixtures (samples A-D) provided by SIAD.

Constituents According to ISO14687:2019	Sample A filling 09.08.2022		Sample B filling 03.03.2023		Sample C filling 03.03.2023		Sample D filling 03.03.2023	
	Value	u	Value	u	Value	u	Value	u
Water (H_2O)	-	-	-	-	-	-	-	-
Nitrogen (N_2)	-	-	-	-	-	-	-	-
Helium (He)	340.000	7.000	303.000	6.200	-	-	-	-
Argon	303.000	7.000	303.000	6.200	-	-	-	-
Oxygen (O_2)	6.700	1.000	4.200	0.400	-	-	-	-
Methane (CH_4)	101.500	4.500	103.000	2.100	-	-	-	-
Propane	1.950	0.100	2.000	0.100	-	-	-	-
Carbon Dioxid (CO_2)	2.090	0.110	2.150	0.110	-	-	-	-
Carbon Monoxid (CO)	0.190	0.200	0.189	0.016	-	-	-	-
Ammonia (NH_3)	1.050	0.200	1.020	0.130	-	-	-	-
COS	0.054	0.005	1.020	0.130	-	-	-	-
Formaldehyde (HCHO)	0.100	0.020	0.054	0.005	0.116	0.040	-	-
Formic acid (HCOOH)	0.200	0.032	0.054	0.005	-	-	0.201	0.032
Trichlorethen (Cl_3C_2H)	0.045	0.005	0.050	0.005	-	-	-	-
Dichlorethane ($Cl_2C_2H_4$)	0.051	0.005	0.053	0.005	-	-	-	-
$Cl_3F_3C_2$ (Freon 113)	0.045	0.005	0.058	0.005	-	-	-	-

Table S2: Detailed breakdown of the production days and analysis days of samples A-D.

		Sample			
	Steps	A	B	C	D
SIAD	Preparation of gas mixture	09.08.2022	03.03.2023	03.03.2023	03.03.2023
	Gas Analysis	27.09.2022	09.05.2023	27.03.2023	07.03.2023
	Transport: IT - AUT	03.10.2022	24.05.2023	24.05.2023	24.05.2023
Analysis: HyCentA	MEA 1	04.10.2022	25.07.2023	26.05.2023	25.05.2023
	MEA 2	05.10.2022	26.07.2023		
	MEA 3	06.10.2022	27.07.2023		
	MEA 4	07.10.2022	28.07.2023		
	MEA 5	11.10.2022	03.08.2023		
	MEA 6	18.10.2022	10.08.2023		
	MEA 7	27.10.2022	17.08.2023		
	MEA 8	03.11.2022	24.08.2023		
	MEA 9	08.11.2022	31.08.2023		
	MEA 10	15.11.2022	07.09.2023		
	MEA 11	16.01.2023	27.09.2023		

Table S3: Calibration standards for EI-IMR-MS calibration provided by Gas Supplier: SIAD, Bergamo, unless otherwise indicated.

Ref. Stand.	Components	µmol/mol	Ref. Stand.	Components	µmol/mol
CalGas 1	Helium	1000	CalGas 6	Carbon monoxide	20
	Nitrogen	2000		Carbon dioxide	5
	Oxygen	50		Hydrogen	Matrix
	Argon	1000	CalGas 7	Ammonia	5
	Hydrogen	Matrix		Hydrogen	Matrix
CalGas2	Benzen (Benzol):	1	CalGas 8	Hydrogen chloride	5
	Toluol	1		Hydrogen	Matrix
	Xylol	1	CalGas 9	Bromoform	3
	Methylmercaptan	1		Perchloroethylene	5
	Hydrogen sulfide	5		Dibromochloromethane	5
	Carbonyl sulfide	1		1.2-dichloroethane	3
	Hydrogen	Matrix		Trichloroethene	3
CalGas 3	Sulfur dioxide	5		Trichloromethane	3
	Hydrogen	Matrix		Dichloromethane	3
CalGas 4	Carbon disulfide	1		Hydrogen	Matrix
	Ethyl mercaptan	1	CalGas 10*	Hydrogen 7.0	
	Ethene	5	CalGas 11	Formaldehyde	5
	Hydrogen	Matrix		Nitrogen	Matrix
CalGas 5	Methan	5	CalGas 12	Formic acid	10
	Acetylenes	1		Nitrogen	Matrix
	Ethan	1			
	Propane	1			
	Butane	1			
	n-Pentane	1			
	Hydrogen	Matrix			

*supplied by Linde

Table S4: Gas standards for enhancement calibrations used for EI-IMR-MS extended calibration.

Molecule to adjust	Interference molecule
Formaldehyde	Ethane
Sulphur dioxide	1.2 Dichloroethane (C2H4Cl2)
Benzene	Carbon disulfide
Carbonyl sulfide	Ethyl mercaptan
Carbon Dioxide	Propane

Table S5: Analyte concentrations of sample A.

Constituents	Method	Unit	RV		MEA 1		MEA 2	
			Value	u	Value	u	Value	u
Water	FTIR	[ppm]	-	-	30.758	0.398	31.338	0.372
Nitrogen	EI MS	[ppm]	-	-	5511.790	10.099	5554.707	49.703
Helium	EI MS	[ppm]	340.000	7.000	357.243	1.313	356.343	1.182
Argon	EI MS	[ppm]	303.000	7.000	282.617	1.611	283.481	1.258
Oxygen	IMR MS	[ppm]	6.700	1.000	9.625	0.338	9.159	0.383
Methane	IMR MS	[ppm]	101.500	4.500	103.632	0.838	104.139	0.922
Propane	IMR MS	[ppm]	1.950	0.100	3.034	0.085	2.969	0.083
Carbon Dioxid	FITR	[ppm]	2.090	0.110	2.180	0.018	2.195	0.016
Carbon Monoxid	FTIR	[ppm]	0.190	0.200	0.212	0.006	0.209	0.006
Ammonia	FTIR	[ppm]	1.050	0.200	0.917	0.026	0.930	0.024
Carbonylsulfid	FITR	[ppm]	0.054	0.005	0.058	0.001	0.059	0.001
Trichlorethen	IMR MS	[ppm]	0.045	0.005	0.053	0.006	0.055	0.010
1.2 Dichloroethane	IMR MS	[ppm]	0.051	0.005	0.061	0.006	0.059	0.005
Freon 113	FTIR	[ppm]	0.045	0.005	0.310	0.005	0.302	0.005
**Constituents**	**Method**	**Unit**	MEA 3		MEA 4		MEA 5	
			Value	u	Value	u	Value	u
Water	FTIR	[ppm]	30.976	0.231	30.942	0.269	31.290	0.231
Nitrogen	EI MS	[ppm]	5430.261	14.881	5551.453	13.390	5555.340	14.881
Helium	EI MS	[ppm]	350.622	1.171	357.058	1.753	357.512	1.171
Argon	EI MS	[ppm]	279.138	0.626	283.926	0.452	284.211	0.626
Oxygen	IMR MS	[ppm]	7.554	0.370	8.534	0.255	8.745	0.370
Methane	IMR MS	[ppm]	102.964	0.517	103.544	0.505	103.389	0.517
Propane	IMR MS	[ppm]	2.895	0.106	2.727	0.074	2.695	0.106
Carbon Dioxid	FITR	[ppm]	2.181	0.011	2.183	0.016	2.177	0.011
Carbon Monoxid	FTIR	[ppm]	0.208	0.005	0.208	0.005	0.208	0.005
Ammonia	FTIR	[ppm]	0.923	0.025	0.911	0.039	0.917	0.025
Carbonylsulfid	FITR	[ppm]	0.059	0.001	0.059	0.001	0.058	0.001
Trichlorethen	IMR MS	[ppm]	0.050	0.012	0.052	0.011	0.053	0.012
1.2 Dichloroethane	IMR MS	[ppm]	0.060	0.006	0.059	0.007	0.057	0.006
Freon 113	FTIR	[ppm]	0.293	0.009	0.304	0.005	0.293	0.009
**Constituents**	**Method**	**Unit**	MEA 4		MEA 5		MEA 6	
			Value	u	Value	u	Value	u
Water	FTIR	[ppm]	30.942	0.269	31.290	0.396	31.366	0.332
Nitrogen	EI MS	[ppm]	5551.453	13.390	5555.340	8.059	5592.912	123.035
Helium	EI MS	[ppm]	357.058	1.753	357.512	1.121	338.645	1.500
Argon	EI MS	[ppm]	283.926	0.452	284.211	0.400	284.071	0.478
Oxygen	IMR MS	[ppm]	8.534	0.255	8.745	0.242	9.430	0.470
Methane	IMR MS	[ppm]	103.544	0.505	103.389	0.711	103.297	0.363
Propane	IMR MS	[ppm]	2.727	0.074	2.695	0.082	2.827	0.061
Carbon Dioxid	FITR	[ppm]	2.183	0.016	2.177	0.012	2.179	0.036
Carbon Monoxid	FTIR	[ppm]	0.208	0.005	0.208	0.007	0.206	0.006
Ammonia	FTIR	[ppm]	0.911	0.039	0.917	0.029	0.898	0.025
Carbonylsulfid	FITR	[ppm]	0.059	0.001	0.058	0.001	0.059	0.001
Trichlorethen	IMR MS	[ppm]	0.052	0.011	0.053	0.017	0.052	0.012
1.2 Dichloroethane	IMR MS	[ppm]	0.059	0.007	0.057	0.006	0.059	0.007
Freon 113	FTIR	[ppm]	0.304	0.005	0.293	0.009	0.303	0.004

Continuation of **Table S5**

**Constituents**	**Method**	**Unit**	MEA 7		MEA 8		MEA 9	
			Value	u	Value	u	Value	u
Water	FTIR	[ppm]	31.410	0.427	31.995	0.338	30.479	0.205
Nitrogen	EI MS	[ppm]	5543.059	12.420	5552.772	21.411	5541.166	8.013
Helium	EI MS	[ppm]	338.368	1.607	339.298	1.649	335.560	0.987
Argon	EI MS	[ppm]	283.930	0.320	283.999	0.330	283.183	0.354
Oxygen	IMR MS	[ppm]	9.048	0.341	10.350	0.840	9.411	0.245
Methane	IMR MS	[ppm]	102.984	0.089	103.367	0.144	103.305	0.158
Propane	IMR MS	[ppm]	2.893	0.075	1.868	0.060	1.707	0.026
Carbon Dioxid	FITR	[ppm]	2.166	0.002	2.179	0.007	2.176	0.005
Carbon Monoxid	FTIR	[ppm]	0.206	0.005	0.208	0.006	0.206	0.005
Ammonia	FTIR	[ppm]	0.886	0.017	0.848	0.040	0.954	0.015
Carbonylsulfid	FITR	[ppm]	0.058	0.001	0.058	0.001	0.058	0.001
Trichlorethen	IMR MS	[ppm]	0.056	0.018	0.057	0.013	0.045	0.002
1.2 Dichloroethane	IMR MS	[ppm]	0.063	0.004	0.058	0.004	0.054	0.002
Freon 113	FTIR	[ppm]	0.298	0.003	0.288	0.006	0.304	0.003
Constituents	Method	Unit	MEA 10		MEA 11		Mean	
			Value	u	Value	u	Value	u
Water	FTIR	[ppm]	32.214	0.310	30.443	0.184	31.201	0.315
Nitrogen	EI MS	[ppm]	5557.582	40.373	5563.931	6.336	5541.361	27.975
Helium	EI MS	[ppm]	338.249	0.672	358.278	0.917	347.925	1.261
Argon	EI MS	[ppm]	283.988	0.364	284.614	0.253	283.378	0.586
Oxygen	IMR MS	[ppm]	10.880	1.070	9.253	0.569	9.272	0.466
Methane	IMR MS	[ppm]	103.534	0.694	100.037	1.440	103.108	0.580
Propane	IMR MS	[ppm]	1.869	0.042	1.804	0.055	2.481	0.068
Carbon Dioxid	FITR	[ppm]	2.188	0.014	2.067	0.037	2.170	0.016
Carbon Monoxid	FTIR	[ppm]	0.211	0.006	0.201	0.008	0.208	0.006
Ammonia	FTIR	[ppm]	0.936	0.065	1.021	0.068	0.922	0.034
Carbonylsulfid	FITR	[ppm]	0.059	0.001	0.051	0.002	0.058	0.001
Trichlorethen	IMR MS	[ppm]	0.052	0.009	0.049	0.006	0.052	0.011
1.2 Dichloroethane	IMR MS	[ppm]	0.058	0.008	0.059	0.005	0.059	0.005
Freon 113	FTIR	[ppm]	0.304	0.002	0.274	0.007	0.298	0.005

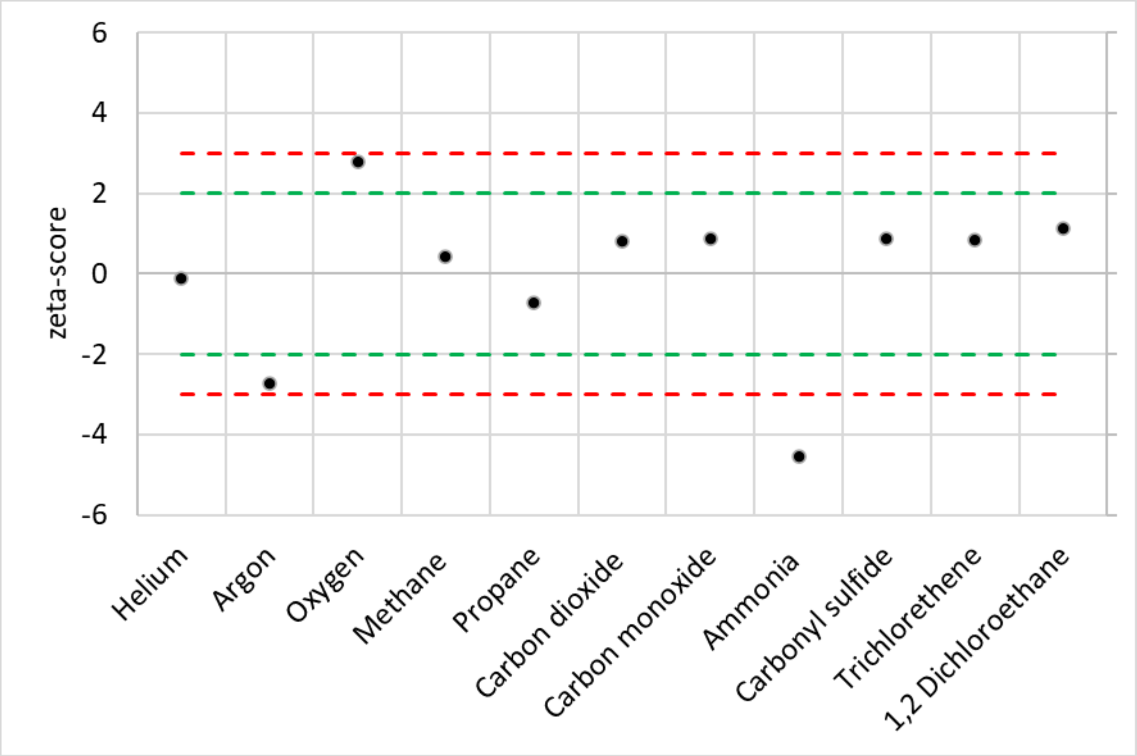

Figure S1: Zeta-score evaluation of sample A, showing a satisfactory zeta score of -0.70 for propane 86 days after gas mixing (MEA 8).

Table S6: Analyte concentrations of sample A.

Constituents	Method	Unit	CV		MEA 1		MEA 2	
			Value	u	Value	u	Value	u
Helium	EI MS	[ppm]	303.000	6.200	313.311	2.486	314.631	2.184
Argon	EI MS	[ppm]	303.000	6.200	309.152	0.846	309.247	0.628
Oxygen	EI MS	[ppm]	4.200	0.400	5.556	0.521	4.546	0.469
Methane	IMR MS	[ppm]	103.000	2.100	102.388	1.132	103.885	1.169
Methane	FTIR	[ppm]	103.000	2.100	109.702	0.214	109.846	0.613
Propane	MS	[ppm]	2.000	0.100	1.940	0.050	1.929	0.049
Carbon Dioxide	FTIR	[ppm]	2.150	0.110	2.236	0.005	2.236	0.014
Carbon Dioxide	MS	[ppm]	2.150	0.110	2.443	0.131	2.166	0.134
Carbon Monoxide	FTIR	[ppm]	0.189	0.016	0.212	0.006	0.210	0.007
Ammonia	MS	[ppm]	1.020	0.130	0.826	0.063	0.866	0.041
Ammonia	FTIR	[ppm]	1.020	0.130	0.892	0.327	0.965	0.513
Carbonyl Sulfide	MS	[ppm]	0.054	0.005	0.084	0.003	0.082	0.002
Carbonyl Sulfide	FTIR	[ppm]	0.054	0.005	0.060	0.001	0.060	0.001
Trichloroethylene	MS	[ppm]	0.050	0.005	0.051	0.005	0.046	0.004
1.2 Dichloroethane	MS	[ppm]	0.053	0.005	0.060	0.006	0.057	0.008
Freon 113	FTIR	[ppm]	0.058	0.005	0.057	0.011	0.063	0.004

Constituents	Method	Unit	MEA 3		MEA 4		MEA 5	
			Value	u	Value	u	Value	u
Helium	EI MS	[ppm]	293.706	5.497	311.434	2.610	313.757	4.118
Argon	EI MS	[ppm]	310.729	2.591	307.376	1.323	308.537	1.150
Oxygen	EI MS	[ppm]	6.038	0.572	9.397	1.802	5.481	0.723
Methane	IMR MS	[ppm]	105.013	1.749	104.675	1.297	105.535	1.412
Methane	FTIR	[ppm]	109.432	0.102	109.540	0.075	109.773	0.082
Propane	MS	[ppm]	1.994	0.043	1.879	0.060	1.981	0.034
Carbon Dioxide	FTIR	[ppm]	2.220	0.005	2.227	0.002	2.238	0.007
Carbon Dioxide	MS	[ppm]	2.406	0.122	2.496	0.117	2.408	0.111
Carbon Monoxide	FTIR	[ppm]	0.206	0.006	0.208	0.005	0.212	0.005
Ammonia	MS	[ppm]	0.857	0.081	0.851	0.070	0.846	0.068
Ammonia	FTIR	[ppm]	1.050	0.176	0.913	0.406	1.068	0.072
Carbonyl Sulfide	MS	[ppm]	0.087	0.003	0.086	0.004	0.085	0.003
Carbonyl Sulfide	FTIR	[ppm]	0.060	0.001	0.060	0.001	0.060	0.001
Trichloroethylene	MS	[ppm]	0.049	0.005	0.048	0.006	0.048	0.004
1.2 Dichloroethane	MS	[ppm]	0.057	0.006	0.056	0.005	0.056	0.006
Freon 113	FTIR	[ppm]	0.061	0.011	0.066	0.006	0.057	0.003

Continuation of **Table S6**

Constituents	Method	Unit	MEA 6 Value	MEA 6 u	MEA 7 Value	MEA 7 u	MEA 8 Value	MEA 8 u
Helium	EI MS	[ppm]	311.222	2.264	316.256	5.135	324.535	5.670
Argon	EI MS	[ppm]	307.161	1.397	308.928	2.364	316.849	3.631
Oxygen	EI MS	[ppm]	9.182	1.472	6.572	0.589	5.024	2.535
Methane	IMR MS	[ppm]	104.834	1.434	104.322	1.002	103.111	1.710
Methane	FTIR	[ppm]	109.531	0.060	109.366	0.110	109.633	0.072
Propane	MS	[ppm]	1.882	0.060	1.909	0.062	1.825	0.054
Carbon Dioxide	FTIR	[ppm]	2.230	0.002	2.235	0.004	2.231	0.003
Carbon Dioxide	MS	[ppm]	2.509	0.131	2.503	0.182	0.000	0.000
Carbon Monoxide	FTIR	[ppm]	0.210	0.007	0.252	0.006	0.209	0.006
Ammonia	MS	[ppm]	0.863	0.072	0.898	0.043	0.898	0.043
Ammonia	FTIR	[ppm]	0.987	0.280			1.097	0.144
Carbonyl Sulfide	MS	[ppm]	0.087	0.004	0.085	0.003	0.051	0.003
Carbonyl Sulfide	FTIR	[ppm]	0.060	0.001	0.058	0.001	0.060	0.001
Trichloroethylene	MS	[ppm]	0.048	0.005	0.048	0.005	0.051	0.004
1.2 Dichloroethane	MS	[ppm]	0.055	0.005	0.055	0.006	0.056	0.006
Freon 113	FTIR	[ppm]	0.057	0.005	0.062	0.004	0.071	0.007

Constituents	Method	Unit	MEA 9 Value	MEA 9 u	MEA 10 Value	MEA 10 u	Mean Value	Mean u
Helium	EI MS	[ppm]	318.722	1.909	321.412	2.045	313.898	3.392
Argon	EI MS	[ppm]	309.984	0.481	312.357	0.386	310.032	1.480
Oxygen	EI MS	[ppm]	5.171	0.486	6.080	0.327	6.305	0.950
Methane	IMR MS	[ppm]	102.270	1.463	104.851	0.994	104.088	1.336
Methane	FTIR	[ppm]	109.564	0.140	109.687	0.145	109.607	0.161
Propane	MS	[ppm]	1.958	0.087	1.995	0.072	1.929	0.057
Carbon Dioxide	FTIR	[ppm]	2.232	0.004	2.237	0.004	2.232	0.005
Carbon Dioxide	MS	[ppm]	0.753	0.112	0.812	0.123	1.850	0.116
Carbon Monoxide	FTIR	[ppm]	0.207	0.006	0.208	0.006	0.213	0.006
Ammonia	MS	[ppm]	0.861	0.082	0.901	0.041	0.867	0.060
Ammonia	FTIR	[ppm]	1.091	0.187	1.134	0.072	1.155	0.242
Carbonyl Sulfide	MS	[ppm]	0.056	0.002	0.057	0.002	0.076	0.003
Carbonyl Sulfide	FTIR	[ppm]	0.060	0.001	0.060	0.001	0.060	0.001
Trichloroethylene	MS	[ppm]	0.048	0.006	0.047	0.005	0.048	0.005
1.2 Dichloroethane	MS	[ppm]	0.054	0.006	0.055	0.006	0.056	0.006
Freon 113	FTIR	[ppm]	0.059	0.011	0.059	0.011	0.061	0.007

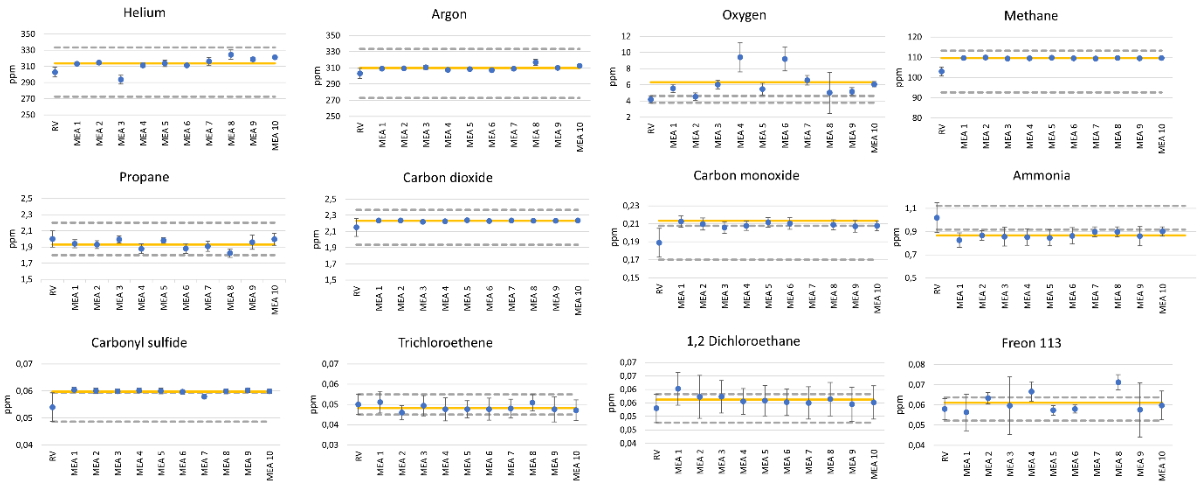

Figure S2: Comparison of reference values (RV) and observed analyte concentrations of sample B at different time points (MEA1-10) in the 208-day stability investigation.
